# Virtual Compared to In-Person Obstetric Anesthesiology Trainee Education During the COVID-19 Pandemic: A Short Report

**DOI:** 10.7759/cureus.26423

**Published:** 2022-06-29

**Authors:** Kelly Fedoruk, Gillian Abir, Brendan Carvalho

**Affiliations:** 1 Anesthesiology, Stanford University, Stanford, USA

**Keywords:** covid-19, obstetric, medical education, virtual education, obstetric anesthesiology

## Abstract

Background: The COVID-19 pandemic brought many changes to medical training, including in-person education platforms being disbanded and replaced with virtual education. At our institution, dedicated obstetric anesthesiology teaching for residents and fellows occurs daily and is highly valued and rated. In March 2020 due to the COVID-19 pandemic, we changed the teaching platform from in-person to virtual teaching (via video communication). We subsequently surveyed residents, fellows, and attendings to determine the impact of virtual compared with in-person teaching.

Methods: To assess the impact of this change, an electronic survey was sent to 10 anesthesiology residents on their 2nd obstetric anesthesiology rotation, and 10 residents on their 1st rotation, respectively. The electronic survey was also sent to three fellows and eight obstetric anesthesiology attendings. Answers were based on a 5-point Likert scale (1 = strongly disagree, 5 = strongly agree).

Results: The results for 1st rotation residents were higher in all domains compared with 2nd rotation residents and fellows, where “quality” achieved statistical significance (p=0.009) between 1st and 2nd rotation residents. “Engagement” was overall the most impacted domain for trainees. Attendings did not feel that virtual teaching impacted their ability to provide adequate education, however, learner engagement was again the lowest rated domain, and teachers strongly favored resuming in-person teaching.

Conclusions:Virtual teaching is an appropriate alternative when in-person teaching is not possible. Future initiatives are needed to improve engagement and better facilitate virtual teaching.

## Introduction

Didactic and instructive education is a core component of anesthesiology residency training [1]. The coronavirus 2019 disease (COVID-19) pandemic has had a profound impact on medical education and forced a shift toward online education. Procedure-based specialties, such as anesthesiology, have had to adjust standard in-person, hands-on, and technical methods of teaching to comply with physical distancing guidelines to respect the safety and well-being of learners and teachers [2].

Our academic institution’s obstetric anesthesiology rotation involves the education of both junior and senior anesthesiology residents and obstetric anesthesiology fellows. These learners received daily, in-person, and interactive teaching prior to the COVID-19 pandemic. Although we maintained resident and fellow involvement in patient care throughout the pandemic, we pivoted the daily teaching sessions to a virtual education platform via video communication to comply with public health recommendations of physical distancing. To assess the impact this education delivery shift had on the quality of learning and teaching, we surveyed anesthesiology residents, fellows, and attendings on their experience. In particular, the difference between a learner who had rotated on the obstetric anesthesiology service prior to the pandemic and had experienced the standard teaching structure of daily in-person teaching sessions, and those who had not, was assessed.

This article was previously presented as a meeting abstract at the 2021 Society for Obstetric Anesthesia and Perinatology (SOAP) Annual Meeting on May 15, 2021.

## Materials and methods

Description of prior in-person teaching curriculum

Prior to the pandemic, residents scheduled for the obstetric anesthesiology rotation would meet an obstetric anesthesiologist every weekday afternoon to engage in didactic or hands-on learning sessions. Sessions would typically last 1-1.5 hours and include didactic lectures, critical appraisal of literature, case-based discussions, hands-on training of technical procedures, perioperative ultrasound, and low-fidelity simulation. Similar daily teaching occurred to the obstetric anesthesiology fellows. Over the years, these daily teaching sessions have been highly regarded among trainees, as evidenced by repeatedly positive and highly ranked evaluations: “Best part is the dedicated teaching time. It really cements what is seen clinically into our mind and helps us understand the anesthetic choices for obstetrics”; “Overall learning and teaching value is phenomenal”; “One of the best teaching rotations in the residency. Greatly appreciate the daily lectures. Good mix of straightforward and clinically more complex cases.”

Description of the virtual video communication platform for teaching

In March 2020, when physical distancing restrictions were enforced in the United States due to the COVID-19 pandemic, in-person education sessions were facilitated virtually. The daily attending obstetric anesthesiologist was tasked with leading a teaching session via Zoom video communication software (San Jose, CA). There was no defined format prescribed for the style of teaching, but efforts were made to maintain the previously interactive nature of these sessions. The choice of topic was unchanged and derived from a list of important topics that are categorized topics as “essential” and “desired”.

Description of the learner’s and attending anesthesiologist’s surveys

As this change marked a drastic shift in our usual teaching modality, we conducted a quality assurance assessment to determine the impact of this education delivery shift. Residents, fellows, and attendings were sent an electronic survey employing a Likert scale (1 = strongly disagree, 5 = strongly agree). The survey response rate was 80% for residents and 100% for fellows and attendings.

Ten residents who were on their 1st rotation at the time of the virtual teaching implementation were surveyed, along with 10 residents on their 2nd rotation who had experienced both in-person and virtual teaching. To differentiate the experience a resident had based on exposure to in-person obstetric anesthesiology teaching prior to the pandemic, we tailored survey questions to the different groups. Three fellows and eight obstetric anesthesiology attendings were surveyed. All fellows and attendings had participated in in-person education prior to virtual teaching implementation. Survey questions are listed in Table 1.

**Table 1:** Survey questions for in-person teaching compared with virtual teaching

A) For residents who have rotated on obstetric anesthesiology before (with in-person teaching):
1) Compared with in-person teaching, virtual teaching was of equivalent quality?
2) Compared with in-person teaching, virtual teaching provided the same ability to learn?
3) Compared with in-person teaching, virtual teaching provided the same ability for you to stay engaged?
4) Compared with in-person teaching, virtual teaching provided an equivalent level of communication with your presenter?
5) For future residents (when social distancing is disbanded), in-person teaching should resume instead of virtual teaching?
B) For residents who have not rotated on obstetric anesthesiology before:
1) Virtual teaching has been of good quality?
2) Virtual teaching has been easy for you to learn?
3) Virtual teaching has been easy for you to stay engaged?
4) Virtual teaching has been easy for you to communicate with your presenter?
5) For your second obstetric anesthesiology rotation (if social distancing is disbanded), in-person teaching should resume instead of virtual?
C) For obstetric anesthesiology fellows:
1) Compared with in-person teaching, virtual teaching has been equivalent quality?
2) Compared with in-person teaching, virtual teaching provided the same ability to learn?
3) Compared with in-person teaching, virtual teaching provided the same ability for you to stay engaged?
4) Virtual teaching has been easy for you to communicate with your presenter?
5) When social distancing is disbanded, in-person teaching should resume instead of virtual teaching?
D) For obstetric anesthesiology attendings:
1) Compared with in-person teaching, virtual teaching has been equivalent to your ability to deliver teaching material?
2) Compared with in-person teaching, virtual teaching (in your opinion) gave the learners the same ability to learn?
3) Compared with in-person teaching, virtual teaching (in your opinion) was easy for the learners to stay engaged?
4) Compared with in-person teaching, virtual teaching has been easy for you to communicate with your learners?
5) When social distancing is disbanded, in-person teaching should resume instead of virtual teaching?

Statistical Methods

Mean and standard deviation were determined for Likert scores. Survey scores were compared between 1st and 2nd rotation residents using a two-sample t-test. Statistical analysis was performed using Microsoft Excel.

The Stanford University Institutional Review Board determined that this quality improvement project did not meet the definition of human subject research and deemed exempt from requiring ethics approval.

## Results

The group survey results are displayed in Tables 2-3. The results for 1st rotation residents were higher (i.e., more positive) in all domains compared with 2nd rotation residents and fellows. The difference in teaching “quality” achieved statistical significance (p=0.009) between 1st and 2nd rotation residents, along with the desire to return to in-person teaching (p=0.002). The engagement was the most impacted domain for all trainees, in that it achieved the lowest scores across all the residents and fellows surveyed. Attending obstetric anesthesiologists strongly desired the return to in-person teaching when restrictions allowed and also ranked the learner’s ability to stay engaged the lowest.

**Table 2:** Trainee survey results for in-person teaching compared with virtual teaching 5-point Likert scale: 1 = strongly disagree; 5 = strongly agree.

Compared with in-person teaching, virtual teaching…	1^st^ rotation residents (n=8)	2^nd^ rotation residents (n=8)	Difference 1^st^ vs. 2^nd^ rotation residents	Fellows (n=3)
Was of equivalent quality (good quality*)	4.63 (0.52)	3.25 (1.16)	1.38 (0.41, 2.34) p=0.009	3.67 (1.53)
Provided the same ability (easy*) for you to learn	4.13 (0.83)	3.50 (1.20)	0.63 (-0.48, 1.73) p=0.245	4.00 (1.0)
Provided the same ability (easy*) for you to stay engaged	3.88 (0.64)	3.25 (0.71)	0.63 (-0.10, 1.35) p=0.085	2.67 (1.15)
Provided an equivalent level of communication (easy to communicate*) with the presenter	3.88 (0.99)	3.13 (0.83)	0.75 (-0.23, 1.73) p=0.124	3.33 (1.15)
In-person teaching should resume, when able	4.88 (0.35)	3.88 (0.64)	1.0 (0.44, 1.56) p=0.002	4.33 (1.15)

**Table 3:** Attending anesthesiologist’s survey results for in-person teaching compared with virtual teaching

Compared with in-person teaching, virtual teaching…	Attendings (n=8)
Was equivalent in your ability to teach	3.25 (1.16)
Was equivalent in your ability to stay engaged	3.50 (1.41)
Was easy for you to communicate with your learners	3.13 (0.83)
Gave your learners the same ability to learn	3.13 (0.99)
Was easy for your learners to stay engaged	2.75 (0.89)
Was easy for your learners to communicate with you	3.38 (0.74)
In-person teaching should resume, when able	4.00 (0.93)

## Discussion

Residency programs and medical schools all around the world were abruptly challenged with the onset of the COVID-19 pandemic. These challenges arose in multiple domains and included: resident assessment; case exposure and technical procedure logs; simulation and knowledge/skill acquisition [3-5]. Although this disruption will have arguably long-lasting effects on learners’ education experience and overall well-being, it was an opportunity for innovation and ingenuity within the field of medical education and resulted in a collaborative, worldwide effort to educate medical learners in novel ways [6]. At our academic institution, delivering education safely while maintaining the highest standard of quality was our utmost priority.

Virtual education platforms have been increasingly utilized for medical education even prior to the COVID-19 pandemic [7]. The forced transition with the onset of the pandemic provided insight into the experience of learners and educators with this platform. Successful experiences have been documented. A study assessing the quality of virtual education of a heterogeneous group of surgical trainees during the COVID-19 pandemic drew very positive conclusions [8]. Of the 53 responses, 72% felt that engagement had increased in this model of teaching, 88% concluded they were “satisfied” with the virtual platform and 92% concluded the “platform played a pivotal role in helping maintain team morale during this period”. Of note, this study described the use of a different virtual platform than ours and involved a formal and collated lecture series versus the “ad-hoc” nature of teaching our division typically provides. It is worth noting that studies such as this example evaluated a very skilled and experienced group of learners: those in postgraduate medical training. A group that has undoubtedly been greatly impacted by the virtual curriculum is the one with undergraduate medical students who were entering their clinical training at the onset of the pandemic. As an internship or clerkship is often a medical student’s first foray into the practical application of their knowledge and skills, adequately educating via a virtual platform may seem particularly challenging. Hybrid models of delivery in this circumstance have been described, where infectious disease exposure potential was minimized by limiting hospital-based teaching while delivering any possible didactics virtually [9]. Another particularly vulnerable group is the medical learners in low- and middle-income countries (LMIC). International education curriculums were forced to change to not only a virtual, but also a remote international learning experience. One survey described a group of emergency medicine residents in India who participated in such a curriculum. Of interest, they had been receiving in-person education sessions via an outreach education program prior to the pandemic and therefore had this experience to compare. The video lecture series delivered yielded mean and median 10-point Likert scale satisfaction scores of 9.25 and 10, respectively [10]. Though this study concluded a high level of satisfaction, the authors acknowledge that translation into successful clinical performance would be difficult and had not been evaluated.

Our survey was unique in that it sampled learners who had experienced both in-person and virtual teaching methods, and also those whose only experience of our particular education model was virtual in nature. Of interest, the assessment of teaching “quality” in our study was markedly different between the two groups. Residents on their 2nd rotation scored “quality” significantly lower, perhaps because they had favorable previous experience with in-person teaching. Despite this, residents on their 2nd rotation rated the desire to return to in-person teaching lower than those on their 1st rotation. This could reflect on one of the advantages of virtual education: the flexibility in performing it remotely and the potential for greater convenience for the trainee to attend when not at the site of training. Fellows graded “engagement” low, and although attending anesthesiologists scored their own ability to remain engaged relatively high, they scored “learner engagement” lowest of all assessed domains. The low scores given to “learner engagement” are unsurprising due to multiple opportunities for distraction when interacting virtually. Christopoulos et al. have described their experience in increasing learner engagement with virtual platforms and comment that “the more interactive the virtual world is, the higher levels of engagement with the learning activities the learners can reach” [11]. Another recent letter detailed efforts made to orient staff to virtual platform software to prevent technical delays and difficulties and take advantage of the several interactive tools already in place [12]. A “group engagement score” has even been proposed, potentially allowing educators to track student engagement and modify curriculum based on feedback received pertaining to student participation and interactivity [13]. These lend to the potential improvements future virtual curriculums can incorporate and offer methods of monitoring the education as a means of quality assurance.

Although our survey allowed valuable insight into such an abrupt change in teaching methodology brought on by the COVID-19 pandemic, we acknowledge that our study had limitations. These include the small sample size of our group and non-standardized teaching sessions. Similarly, because the teaching sessions were facilitated by different academic faculty, the quality of the sessions may have varied significantly due to the individuals delivering them and may have been influenced by their familiarity with the technology.

## Conclusions

Virtual video education may be a useful alternative in situations where in-person learning is not possible, or as an adjunct or supplement to in-person education, however, efforts are needed to ensure the experience mirrors the quality of in-person didactic teaching. Based on our results, initiatives to enhance “learner engagement” during virtual education should be prioritized. Additionally, optimal methods to teach virtual team training/simulation, and procedures/diagnostics, e.g., point-of-care-ultrasound techniques need to be explored.
